# Cord blood leptin and insulin levels in association with mitochondrial DNA content

**DOI:** 10.1186/s12967-018-1599-z

**Published:** 2018-08-13

**Authors:** Annette Vriens, Michelle Plusquin, Willy Baeyens, Liesbeth Bruckers, Elly Den Hond, Ilse Loots, Vera Nelen, Greet Schoeters, Bram G. Janssen, Tim S. Nawrot

**Affiliations:** 1 Centre for Environmental Sciences, Hasselt University, Agoralaan Building D, 3590 Diepenbeek, Belgium; 2 Department of Analytical and Environmental Chemistry, Vrije Universiteit Brussel, Brussels, Belgium; 3 Interuniversity Institute for Biostatistics and Statistical Bioinformatics, Hasselt University, Hasselt, Belgium; 4 Provincial Institute for Hygiene, Antwerp, Belgium; 5 Faculty of Social Sciences and IMDO-Institute, University of Antwerp, Antwerp, Belgium; 6 Environmental Risk and Health, Flemish Institute for Technological Research (VITO), Mol, Belgium; 7 School of Public Health, Occupational and Environmental Medicine, Leuven University, Leuven, Belgium

**Keywords:** Neonates, Metabolic programming, Mitochondrial DNA content, Metabolic hormones, Insulin, Leptin

## Abstract

**Background:**

The developmental origins of health and disease theory states that a disturbance in the early life environment can contribute to disease risk in later life. Leptin and insulin are anorectic hormones involved in energy homeostasis and are crucial for foetal growth. Disturbances in the levels of these hormones contribute to obesity and diabetes. In adults, altered mitochondrial function is an important hallmark of metabolic disorders, including obesity and diabetes. However, the mitochondrial effects of early life metabolic variation are unexplored. We investigated whether there is an association between metabolic hormones and mitochondrial DNA (mtDNA) content in early life.

**Methods:**

The study included 236 newborns from the FLEHS III birth cohort, Flanders (Belgium). Relative mtDNA content of cord blood leukocytes was determined using quantitative PCR. Cord blood levels of leptin and insulin were determined using immunoassays. We studied the association between these metabolic hormones and mtDNA content using multiple linear regression models, while accounting for covariates and potential confounders.

**Results:**

Leptin and insulin levels were positively associated with cord blood mtDNA content. mtDNA content was respectively 4.49% (95% CI 1.15–7.93; p = 0.008) and 1.60% (95% CI 0.31–2.91; p = 0.02) higher for a interquartile range increase of respectively cord blood leptin and insulin levels. In a sensitivity analysis, we observed that insulin and leptin were independently associated to mtDNA content and that insulin was stronger associated to mtDNA content in boys than in girls.

**Conclusion:**

Neonatal metabolic hormones were associated with cord blood mtDNA content, which suggests that in early life the variation of mtDNA content might accommodate or reflect changes in the metabolic status.

**Electronic supplementary material:**

The online version of this article (10.1186/s12967-018-1599-z) contains supplementary material, which is available to authorized users.

## Background

According to the developmental origins of health and disease theory, a suboptimal perinatal environment can lead to metabolic programming, which may affect one’s susceptibility for disease in adult life [1]. Perinatal metabolic determinants, such as birth weight and maternal BMI, impact the risk of the development of cardiovascular and metabolic disorders in later life which is characterised by a U-shaped association [2–4].

Leptin and insulin are hormones involved in energy homeostasis. Leptin is an anorectic hormone, which is mainly produced by adipocytes and functions in a negative feedback mechanism to regulate adipocyte size, energy intake/expenditure and the metabolism. Leptin concentrations are correlated with the adipose tissue mass and leptin insensitivity is often observed in obesity [5]. During gestation, the placenta produces leptin and leptin plays an important role in foetal development [6]. Insulin is produced by pancreatic β-cells to regulate glucose homeostasis. Peripheral insulin resistance and dysfunction of pancreatic β-cells cause type 2 diabetes, which is among the most prevalent metabolic disorders in humans [7]. During gestation, maternal insulin levels may rise to facilitate maternal fat storage for maternal energy, while maintaining carbohydrates for placental-foetal transport and foetal growth [8, 9]. In the foetus, β-cells are functional from the 10th week, but only become responsive to glucose in the last trimester [10]. Neonatal concentrations of leptin [11] and insulin [12, 13] are correlated with growth measures at birth. As such, they can be reflective of foetal growth and the metabolic status of the newborn. Furthermore, neonatal levels of leptin [14, 15] and insulin [16] may be predictive of the metabolic status in childhood.

Oxidative stress is the imbalance between production of reactive oxygen species (ROS) and antioxidant defence mechanisms resulting in excessive ROS. It is an important feature in the aetiology of disorders such as type 2 diabetes, obesity and cardiovascular disease. Oxidative stress causes damage to mitochondrial macromolecules, hereby affecting mitochondrial function. As such, alterations in mitochondria and mitochondrial function have been observed in relation to metabolic disorders, in which oxidative stress appears to be a key player [17]. Mitochondrial DNA (mtDNA) content can be indicative of mitochondrial (dys)function [18] and was recently suggested as a biomarker for type 2 diabetes in adults [19]. It is hypothesized that early life metabolic variation or challenges may result in foetal programming, which contributes to the predisposition for disease in adulthood. The relation between metabolic variation and mitochondrial function might already be established in early life. Here, we studied cord blood leptin and insulin levels, which reflect metabolic challenges during gestation, in association with cord mtDNA content, an indicator of mitochondrial function, in healthy neonates.

## Methods

### Study population

This study was part of the third cycle of Flemish Environment and Health study (FLEHS), which recruited mother-newborn pairs in six hospitals in Flanders, Belgium to obtain a representative sample of the population [20]. All women that lived at least 5 years in Flanders, who were able to fill out an extensive Dutch questionnaire, who gave birth in one of the participating hospitals, were eligible to participate in the study.

In total, 281 mother-newborn pairs were recruited from November 2013 to November 2014. Of these 281 pairs, we collected sufficient cord blood from 277 participants to obtain DNA for cord blood mtDNA content measurements. Participants with missing quantification of the hormones insulin (n = 9) or leptin (n = 10), or missing information for important covariables (n = 23) were not included in the analyses. Furthermore, we excluded mothers with gestational diabetes and/or insulin medication use, as well as mothers that delivered through caesarean section. In this study, 236 mother-newborn pairs were included (detailed information in flow chart in Additional file 1: Figure S1). The medical ethical committee of University of Antwerp and University Hospital of Antwerp as well as the local ethical committee of each participating hospital approved the study. All subjects in the study gave informed consent to participate.

### Data collection

The mothers filled out a questionnaire addressing their general health status (e.g. gestational weight gain, gestational diabetes, medication use during pregnancy), lifestyle (e.g. smoking during pregnancy), socio-economic status (e.g. occupation, education), household composition & housing conditions and dietary patterns. Information on birth weight, birth length, head circumference and gestational age were obtained from medical records at the maternity ward. Small for gestational age (SGA) was defined as a birth weight below the 10th percentile for gestational age and sex, according to the sex-specific references curves. Similarly large for gestational age (LGA) was defined as a birth weight above the 90th percentile for gestational age and gender. The references were based on data of singleton births in Flanders from the Study Centre for Perinatal Epidemiology in the period 2001–2010 [21].

### Sample collection

Umbilical cord blood was collected immediately after delivery using polypropylene Na-EDTA tubes, which were tested for metal contamination. Blood cell counts were determined on a fresh sample using an automated haematology analyser (HST-N302; Sysmex XE-2100 and SP-1000i). Samples were centrifuged at 3200 rpm for 15 min to retrieve buffy coats for DNA isolation and plasma for the hormone quantification. Samples were stored at − 80 °C until further analyses.

### Determination of the mitochondrial DNA content

DNA was isolated from buffy coats containing cord blood leukocytes using the QIAamp DNA mini kit (Qiagen). Mitochondrial DNA content (mtDNA) in cord leukocytes was measured by determining the ratio of two mitochondrial gene copy numbers (*MTF3212/R3319* and *MT*-*ND1*) to a single-copy nuclear control gene (*RPLP0*) using a real-time quantitative polymerase chain reaction (qPCR). qPCR reactions were carried out in triplicate on a 384-well plate on the 7900HT Fast Real-Time PCR System (Applied Biosystems) in a 10 µl volume containing: 5 µl QuantiTect SYBR Green (Qiagen) mastermix, 0.3 µl of forward and reverse primers (300 nM) and 1.9 µl RNAse-free water and 6 ng DNA diluted in 2.5 µl RNAse-free water. Primer sequences for mitochondrial genes are reported elsewhere [22]. Six interrun calibrators and no-template controls were included in each qPCR run. The thermal cycling profile for the three transcripts was 10 min at 95 °C for activation of the polymerase enzyme and initial denaturation, followed by 40 cycles of 15 s at 94 °C for denaturation and 70 s at 58 °C for annealing and extension. After thermal cycling, the raw data were collected and processed using SDS 2.3 software (Applied Biosystems). The cycle quantification (Cq) values were normalized relatively to the *RPLP0* gene using qBase + software (Biogazelle) taking into account the run-to-run differences [23].

### Quantification of metabolic hormones in cord blood

Leptin and insulin levels were determined in cord blood plasma. Leptin was quantified using a radio-immuno assay (Human-Leptin-RIA-CT (KIPMR44); DIAsource ImmunoAssays). Insulin was measured using a chemiluminescence microparticle immuno assay [Architect Insulin Reagent Kit (8K41); Abbott Laboratories] carried out on a Abbott Architect i2000sr analyser. For successful quantification of insulin, plasma samples were frozen within 6 h after collection.

### Statistical analysis

Data management and statistical analysis was done using SAS software (version 9.4; SAS Institute Inc.). mtDNA content, insulin and leptin were log_10_-transformed to normalize their distribution. Continuous variables are presented as arithmetic means [standard deviation (SD)] or in case of insulin and leptin represented by geometric means (25th–75th percentile). Categorical variables are presented as numbers (frequency in percentage).

We performed univariate linear regression models to assess the associations between potential important covariates and the hormone levels or mtDNA content. The effect estimates of the univariate associations are represented as the mean percentage change (standard error) in respectively mtDNA content (see Table 1) and hormone levels (see Table 2), compared to a reference group for the categorical variables or for a standard deviation increase in the continuous variables.

**Table 1:** Characteristics of the study population (n = 236) and their association with cord blood mtDNA content

Characteristic	Mean (SD) or number (%)	Mean effect (SE)	p-value
Maternal characteristics			
Maternal age, years	30.2 (4.1)	1.23% (1.63)	0.45
Maternal pre-pregnancy BMI, kg/m^2^	23.7 (4.1)	− 1.71% (1.65)	0.30
Maternal pre-pregnancy BMI			
< 18.5 kg/m^2^	7 (2.5%)	10.21% (11.08)	0.36
18.5–25 kg/m^2^	163 (69.1%)	Ref	–
25–30 kg/m^3^	46 (19.5%)	0.82% (4.31)	0.85
> 30 kg/m^2^	21 (8.9%)	− 1.21% (6.03)	0.84
Smoking during pregnancy	23 (9.8%)	7.79% (5.67)	0.17
Highest educational level in household			
Lower high school	17 (7.2%)	4.82% (6.66)	0.47
Higher high school	65 (27.5%)	− 0.15% (3.80)	0.97
College/University	154 (65.3%)	Ref	–
Parity^a^			
0 children	105 (44.5%)	Ref	–
1 child	88 (37.3%)	7.96% (3.68)	0.04
≥ 2 children	43 (18.2%)	7.04% (4.63)	0.13
Newborn characteristics			
Boys	121 (51.3%)	5.96% (3.32)	0.08
Ethnicity^b^			
Belgian	172 (72.9%)	Ref	–
European	20 (8.5%)	3.41% (6.05)	0.57
Non-European	44 (18.6%)	12.42% (4.29)	0.01
Gestational age, weeks	39.3 (1.3)	− 5.45% (1.67)	0.001
Birth weight, grams	3449 (429)	− 1.11% (1.64)	0.50
Large for gestational age	26 (11%)	0.98% (5.42)	0.85
Small for gestational age	19 (8%)	− 3.58% (6.26)	0.55
Leptin, µg/l^c, d^	5.6 (3.4–9.3)	1.38% (1.50)	0.36
Insulin, pmol/l^c, d^	27.3 (18–40.5)	1.94% (0.62)	0.002

The mean (SE) effects are represented as a  % change in the cord blood mtDNA content for a SD change in the continuous variable or compared to a reference category for the class variables

^a^Parity is indicated based on the number of children before the child in our study

^b^Ethnicity was defined by country of birth of the grandparents of the child

^c^For leptin and insulin the geometric mean (25th–75th percentile) is given

^d^Effect sizes for a interquartile range increase in the mean hormone levels

**Table 2:** Correlates of cord blood leptin and insulin levels

	Leptin		Insulin	
	% change (SE)	p-value	% change (SE)	p-value
Maternal characteristics				
Maternal age, years	8.4% (4.4)	0.06	3.8% (4.2)	0.36
Maternal pre-pregnancy BMI, kg/m^2^	13.6% (4.4)	0.002	5.6% (4.2)	0.19
Maternal pre-pregnancy BMI				
< 18.5 kg/m^2^	− 38.5% (31.9)	0.08	14.9% (30.6)	0.60
18.5–25 kg/m^2^	Ref	–	Ref	–
25–30 kg/m^3^	24.3% (11.7)	0.05	17.7% (11.3)	0.13
> 30 kg/m^2^	38.9% (16.7)	0.03	14.7% (16.1)	0.36
Smoking during pregnancy	− 9.5% (16)	0.50	− 3.8% (15.2)	0.78
Highest educational level in the family				
Lower high school	− 6.4% (18.9)	0.70	25% (17.8)	0.18
Higher high school	− 8.1% (10.6)	0.40	7.7% (10)	0.43
College/University	Ref	–	Ref	–
Parity				
0 children	Ref	–	Ref	–
1 child	− 2.2% (10.3)	0.82	6.1% (9.7)	0.52
≥ 2 children	− 2.1% (13.1)	0.86	25.8% (12.3)	0.05
Newborn characteristics				
Boys	− 37% (8.7)	<0.0001	− 11.8% (8.7)	0.13
Ethnicity				
Belgian	Ref	–	Ref	–
European	0.3% (17.4)	0.98	10.1% (16.2)	0.52
Non-European	2.3% (12.2)	0.84	34.7% (11.3)	0.01
Gestational age, weeks				
Birth weight, grams	22% (4.2)	0.03	9.3% (4.2)	0.03
Large for gestational age	68.2% (14.6)	0.0002	39.7% (13.9)	0.01
Small for gestational age	− 23.5% (17)	0.09	− 34.6% (16.2)	0.01

The mean (SE) effects are represented as a relative % change in the cord blood hormone levels for a SD change in the continuous variable or compared to a reference category for the class variables

We then first evaluated the association between mtDNA content and the metabolic hormones with Pearson and Spearman correlation coefficients and we built multiple linear regression models to account for covariates and potential confounders, including cord blood thrombocyte counts, gestational age, newborns’ sex, growth rate (normal, LGA, SGA), maternal pre-pregnancy BMI, maternal age, smoking during pregnancy (yes, no), parity (0, 1, ≥ 2), ethnicity (Belgian, European ancestry, non-European ancestry) and the highest educational level of the household (low, middle, high). The a priori chosen covariates were included in the model regardless of the p-value. Q–Q plots of the residuals were checked to test the assumptions of all linear models. The effect-estimates were calculated as the relative percentage change in mtDNA content associated with an interquartile range (from the 25th to the 75th percentile) increase in cord blood leptin and insulin levels (log10), which corresponds to a 28.1% difference in insulin and a 84.5% difference in leptin.

In a sensitivity analysis, we examined the associations between cord blood mtDNA content and the metabolic hormones in the same model, to indicate if the effects were independent of each other. Furthermore, we evaluated effect modification of the growth rate (SGA, normal, LGA), newborns’ sex and maternal BMI. Lastly, we performed sensitivity analyses in which we excluded mothers with a BMI above 30 kg/m^3^. The results for the associations of mtDNA content and other metabolic factors, including maternal pre-pregnancy BMI, birth weight and growth rate, were provided as Additional file 1: Table S1.

## Results

### Population characteristics

An overview of the population characteristics is presented in Table 1. On average (standard deviation, SD), the mothers were 30.2 (4.1) years old and had a normal pre-pregnancy BMI of 23.7 (4.1) kg/m^2^. 19.5% of the mothers were overweight and 8.9% was obese. 9.8% of the mothers reported smoking during the pregnancy. The majority of the households had a high education level (65.3%) and for 44.5% of the mothers, this newborn was their first child. 51.3% of the newborns were boys, and the birth weight was on average 3449 (429) grams. 11% of the newborns was LGA and 8% of the newborns was SGA. The geometric mean cord blood insulin and leptin levels were respectively 27.3 (25th–75th percentile: 18–40.5) pmol/L and 5.6 (25th–75th percentile: 3.4–9.3) µg/L.

### Correlates of cord blood mtDNA content

Of the aforementioned variables, we assessed univariate associations to determine if they are relevant predictors of cord blood mtDNA content (Table 1). Compared with mtDNA levels of first-born neonates, mtDNA levels were respectively 7.96% (± 3.68%) higher in neonates who were the second (p = 0.04) child in the family. Neonates from non-European ancestry had 12.42% (± 4.29%) higher cord blood mtDNA copy numbers (p = 0.01). A 1.3 week longer gestational age was associated with 5.45% lower (± 1.67%) mtDNA content in cord blood (p = 0.001).

### Correlates of metabolic hormones

In unadjusted analyses, birth weight and growth rate were determinants of both cord blood leptin and insulin levels (Table 2). An SD increase in birth weight (429 grams) was associated with 22% (± 4.2%) higher leptin levels (p = 0.03) and 9.3% (± 4.2%) higher insulin levels (p = 0.03). Compared with newborns with a normal growth rate, LGA babies had 68.2% (± 14.6%) higher leptin levels (p = 0.0002) and 39.7% (± 13.9%) higher insulin levels (p = 0.01). Similarly, SGA babies had on average 23.5% (± 17%) lower leptin levels (p = 0.09) and 34.6% (± 16.2%) lower insulin levels (p = 0.01). Furthermore, parity was associated with insulin levels (overall p = 0.14, difference between 0 and ≥ 2 children p = 0.05). Maternal pre-pregnancy BMI and newborn’s sex were associated with cord leptin levels (p = 0.002). Each SD increase in pre-pregnancy BMI (4.1 kg/m^2^) was associated with 13.6% (± 4.4%) higher leptin (p = 0.004) in cord blood. Boys had on average 38.38% (± 8.48%) lower levels of cord blood leptin (p = 0.002). Compared with newborns of Belgium ethnicity, newborns with non-European ancestry had 34.7% (± 11.3%) higher cord blood insulin concentrations (p = 0.01).

### Metabolic hormones in association with cord blood mtDNA content

**Fig. 1:**
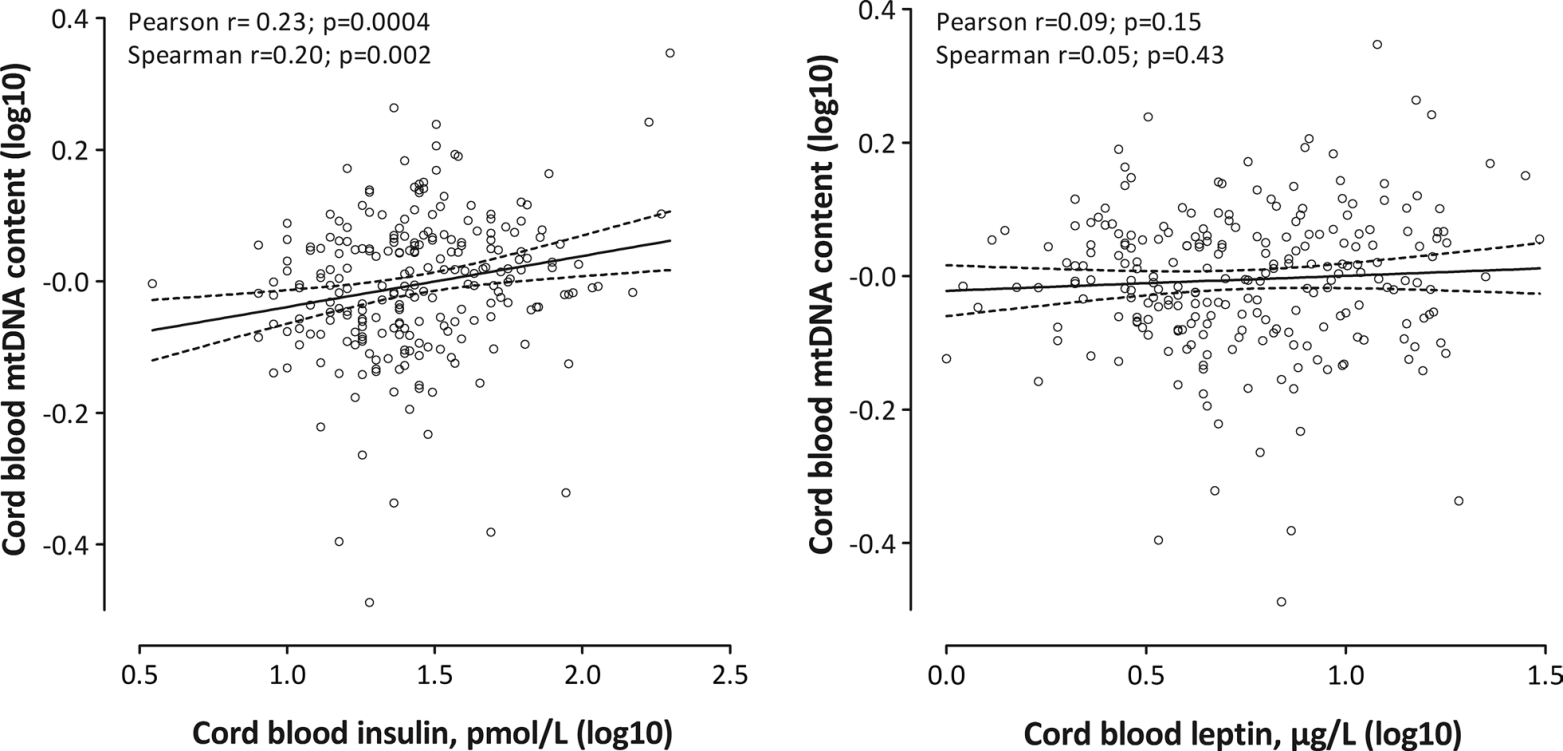
Correlation plots between mtDNA content in cord blood and metabolic hormones (n = 236)

In an unadjusted analysis (Fig. 1), we observed a positive correlation between cord blood insulin and mtDNA content (Pearson r = 0.23, p = 0.0004; Spearman r = 0.20, p = 0.002), contrary cord blood leptin and mtDNA content were not correlated (Pearson r = 0.09, p = 0.15; Spearman r = 0.05, p = 0.43). For an interquartile range increase in insulin levels, we observed a 1.94% (± 0.62%; p = 0.002) higher mtDNA content in cord blood (Table 1).

After adjustment for sex, gestational age, growth rate, ethnicity, maternal pre-pregnancy BMI, maternal age, parity, smoking status during pregnancy, education and thrombocyte count, leptin and insulin levels were significantly associated with cord blood mtDNA content (Table 3). An interquartile range increase in the cord blood leptin levels was associated with a 4.49% (95% CI 1.15–7.93%) higher mtDNA content in cord blood (p = 0.008). Similarly, a interquartile range increase in the mean insulin levels was associated with a 1.60% (95% CI 0.31–2.91%) increase in mtDNA content (p = 0.02). Other metabolic factors were not associated with cord blood mtDNA content (Additional file 1: Table S1).

**Table 3:** The association between metabolic hormones and mitochondrial DNA content

	Effect size (95% CI)	p-value
Main results: separate models for metabolic hormones (n = 236)		
Insulin	1.60% (0.31–2.91)	0.02
Leptin	4.49% (1.15–7.93)	0.008
Sensitivity: combined model including both metabolic hormones (n = 236)		
Insulin	1.29% (− 0.02–2.61)	0.05
Leptin	3.77% (0.40–7.25)	0.03
Sensitivity: exclusion of obese mothers (n = 215)		
Insulin	1.72% (0.37–3.09)	0.01
Leptin	4.75% (1.25–8.37)	0.008
Sensitivity: effect modification* of newborns’ sex for the relation between mtDNA content and insulin		
Girls	0.57% (− 1.19–2.36)	0.53
Boys	1.61% (0.32–2.91)	0.01

The estimated effects (95% CI) are represented as a relative % change in the mtDNA content for a interquartile range increase in hormone levels. Models were adjusted for newborns’ sex, gestational age, growth rate, ethnicity, maternal pre-pregnancy BMI, maternal age, household education, parity, smoking during pregnancy and cord blood thrombocyte count

* p for interaction term = 0.10

In sensitivity analyses, we tested the hormones in the same model, indicating that the effects of insulin and leptin on mtDNA were independent (Table 3). Exclusion of obese (BMI > 30 kg/m^3^) mothers did not affect our results. Furthermore, effect modification of newborn’s sex, growth rate or maternal pre-pregnancy BMI was evaluated. The interaction terms were not significant for growth rate, pre-pregnancy BMI and for newborn’s sex and leptin. Only, the effect of insulin on mtDNA content was different for boys and girls, as shown by a trend of the interaction between insulin and newborn’s sex (p = 0.10). The association between mtDNA content and insulin was more pronounced in boys, while it was not significant in girls (Table 3, Additional file 1: Figure S2).

## Discussion

Early life molecular markers implicated in metabolic programming are widely unexplored. In adults, altered mitochondrial function is a hallmark in the aetiology of cardio-metabolic disorders [17]. In this study, we showed that metabolic hormones and mtDNA content are associated in newborns, which suggests that mtDNA content might reflect or accommodate metabolic variation from early life onwards.

Early life metabolic disturbances may affect mitochondrial function and these effects can persist into adulthood. Experimental evidence showed that long-term adaptations in mitochondrial function arise as a consequence of malnutrition during early life [24–27]. The following epidemiological studies further support a putative role of the mitochondria in programming as a consequence of metabolic challenge. First, placental oxidative stress and mitochondrial dysfunction was observed in maternal obesity coincided with gestational diabetes and correlated with neonatal leptin levels [28]. Also, Clemente et al. reported a positive association between placental mtDNA content and birth weight [29]. In newborns, associations were shown between the mtDNA content and foetal growth rate, showing lower umbilical cord mtDNA content in small for gestational age and large for gestational age neonates [30]. However, in the latter study, the authors did not find an association between mtDNA content and leptin levels [30]. In addition, in a population of obese children between 2 and 18 years old, mtDNA content was positively correlated with BMI [31]. Skeletal muscle mitochondrial oxidative phosphorylation function was measured by phosphocreatine recovery time after exercise measured using ^31^P-MRS, as an indicator of ATP synthesis in healthy children of 8–18 years old [32]. Phosphocreatine recovery time was associated with insulin sensitivity, HDL and triglyceride levels and with the resting energy expenditure [32]. Furthermore, a recent study in 12–19 years old healthy children studied muscle mitochondrial function by means of indicators of the ATP synthesis quantified using ^31^P-MRS. They reported an association between the mitochondrial function and triglyceride to HDL ratio, but not with insulin sensitivity [33]. Taken together, these studies demonstrate that alterations in mtDNA content and the mitochondrial energy production might be indicative of the metabolic phenotype, already in early life without the presence of morbidities. A recent study by Clemente et al. provided evidence that placental mtDNA is associated with infant height at 6 months of age, suggesting that neonatal mtDNA content can be predictive of infant growth [34]. This finding further supports our hypothesis that the relation between mitochondrial function and metabolic balance might already be established from birth onwards.

Perinatal metabolic challenges are linked to oxidative stress, which is a key factor affecting the mitochondria. Luo et al. [35] studied the relationship between markers for oxidative stress (lipid peroxidation) and foetal metabolic factors (insulin, IGF1, IGF2, leptin, adiponectin and ghrelin as markers for foetal growth, insulin sensitivity and energy regulation). The authors reported a negative association between maternal and foetal oxidative stress indicators and the foetal concentrations of ghrelin. These results suggest that metabolic programming may occur in healthy neonates [35]. Furthermore, oxidative stress increases with foetal growth retardation and/or malnutrition, which was exemplified by (i) a study on small for gestational age neonates from malnourished mothers [36] and (ii) differences in the oxidative balance were observed in small for gestational age neonates from mothers without nutritional problems [37]. Our study adds to this evidence that even in a normal physiological range of neonatal concentrations of the metabolic hormones leptin and insulin, variation is associated with the mtDNA content, which may imply foetal metabolic programming. However, we observed no significant association between the maternal BMI or birth weight, other metabolic parameters and mtDNA content (Table 1 and Additional file 1: Table S1). A discrepancy with other studies showing an association between placental mtDNA content and birth weight [29] or between cord blood mtDNA and growth rate [30], might be due to differences in the populations, to difference in the biological samples used, to difference in statistical power or due to differences in residual confounding. Clemente et al. [29] had a much larger sample (926 subjects) and studied placental mtDNA content while we focused on cord blood mtDNA content. While the study of Gemma et al. [30] had a smaller sample, they had a higher portion of newborns with an abnormal weight for gestational age (12.5% LGA and 19.3% SGA) compared with our study.

Since prenatal stressors have an impact on the future health of the newborns, physiological metabolic variation, as exemplified by metabolic hormone levels, can be linked with predisposition to risk factors for disease. Neonatal levels of these hormones are sensitive to metabolic challenges. Cord blood levels of leptin are positively associated with birth weight and may explain up to 22% of the variation in birth weight [11]. Lower leptin levels are linked to more rapid growth (or “catch-up growth”) in early life [15]. Serum leptin levels in early childhood (at 12 months) are associated with a slower foetal growth [38] and a higher increase in BMI in the first 4 months of life [38, 39]. Leptin levels in adulthood are associated inversely to birth weight [39] and are related to obesity [40]. Neonatal leptin levels are higher when gestational diabetes mellitus occurred during pregnancy [41–43] and when mothers had a higher BMI [44]. Leptin levels are also associated with neonatal weight and fat mass [11, 45]. Similar, neonatal insulin levels are correlated with maternal BMI [46]. birth weight as well as growth rate were associated with cord blood leptin and/or insulin levels. Also in our population of healthy neonates, the insulin and leptin levels reflect metabolic variation.

We observed more marked effects of insulin on mtDNA content in boys than in girls. Sex differences in the mitochondrial function and oxidative capacity have been previously described (reviewed in [47]). Animal studies indicate that in female rodents mitochondrial function is more specific and efficient, and less oxidative damage occurs [47]. Accordingly, mitochondria might be better protected in females, which may explain why we observe more marked effects in boys.

The study has some limitations. We only studied mtDNA content, leptin and insulin, however to study the metabolic variation and associated mitochondrial function considering more markers would be advantageous. Furthermore, we cannot exclude reverse causality or discuss the temporal relationships in this study. In other words, we do not know if higher levels of the metabolic hormones affect the mtDNA, or if it is the other way around that altered mtDNA content, as a reflection of mitochondrial function, influences the levels of the metabolic hormones.

## Conclusion

Our study is the first to evaluate mtDNA content in relation to metabolic hormones in healthy neonates. mtDNA content reflects the metabolic variation demonstrated by foetal leptin and insulin concentrations. These findings indicate that the association between mitochondria and the metabolic status might be established at birth. Furthermore, our results contribute to the hypothesis that mitochondria may play an important role in foetal metabolic programming.

Nevertheless, it remains unclear if variation in the mtDNA content is a consequence or the cause of the metabolic status. Longitudinal studies are needed to provide insight in the role of mitochondria in relation to early life metabolic changes and the potential role in programming of later life disease.

## Additional file

**Additional file 1: Figure S1.** Flow chart on the included observations. **Table S1.** The association between metabolic factors and mitochondrial DNA content. **Figure S2.** Correlation plot of mtDNA content and insulin levels, for boys and girls separately.

## Abbreviations

^31^P-MRS: ^31^phosphorus magnetic resonance spectroscopy
ATP: adenosine triphosphate
BMI: body mass index
CI: confidence intervals
FLEHS: Flemish Environment and Health Study
HDL: high density lipoprotein
IGF1: insulin growth factor 1
IGF2: insulin growth factor 2
mtDNA: mitochondrial DNA content
Na-EDTA: sodium ethylenediaminetetraacetic acid
PCR: polymerase chain reaction
ROS: reactive oxygen species
SD: standard deviation
SE: standard error
