# Supplementary material for: Cord blood leptin and insulin levels in association with mitochondrial DNA content
Source: J Transl Med. 2018 Aug 13;16:224. doi: 10.1186/s12967-018-1599-z (PMC6090601; doi:10.1186/s12967-018-1599-z)
Supplement: Supplementary file 1 — Additional file 1: Figure S1. Flow chart on the included observations. Table S1. The association between metabolic factors and mitochondrial DNA content. Figure S2. Correlation plot of mtDNA content and insulin levels, for boys and girls separately. [file 12967_2018_1599_MOESM1_ESM.docx]

**Cord blood leptin and insulin in association with mitochondrial DNA content in newborns**

**Additional information**

A Vriens, M Plusquin, W Baeyens, L Bruckers, E Den Hond, I Loots, V Nelen, G Schoeters, BG Janssen, TS Nawrot

Content:

Figure S1: Flow chart on the included observations.

Table S1: The association between metabolic factors and mitochondrial DNA content.

Figure S2: Correlation plot of mtDNA content and insulin levels, for boys and girls separately.


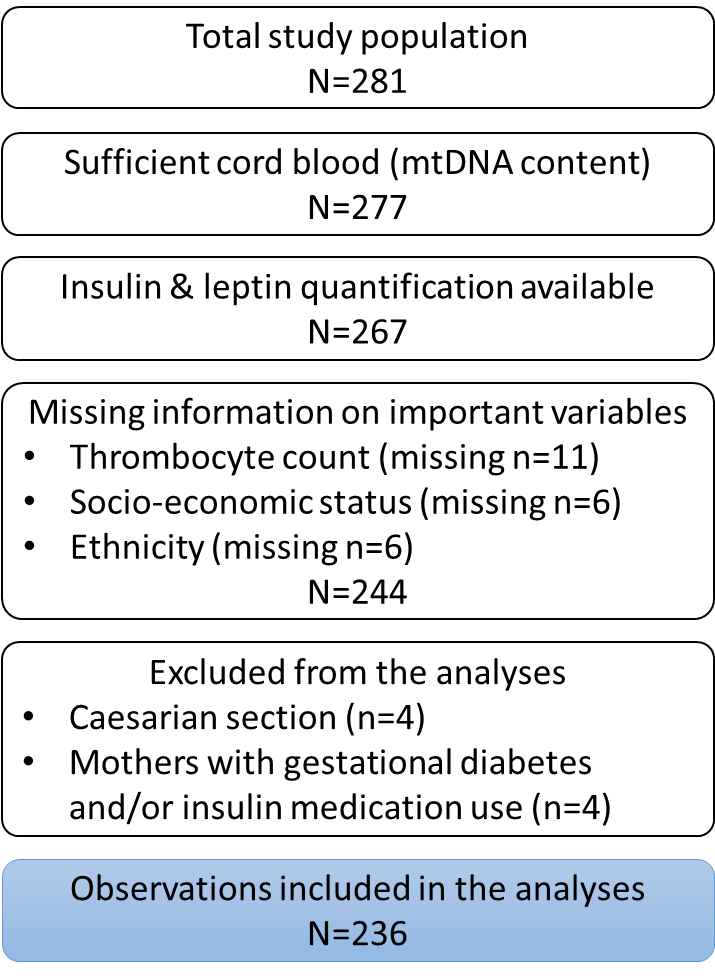


**Figure S1:** Flow chart of data availability and observations in the analyses.

**Table S1:** The association between metabolic factors and mitochondrial DNA content.

|  | **Effect size (95% CI)** | **p-value** |
| --- | --- | --- |
| Maternal pre-pregnancy BMI, + 4.1 kg/m² | -1.30% (-4.59 – 2.02) | 0.44 |
| Birth weight, + 429 grams | 0.68% (-2.92 – 4.27) | 0.71 |
| Growth rate |  |  |
| Small for gestational age | -1.94% (-12.85 – 10.35) | 0.74 |
| Appropriate for gestational age | Ref | - |
| Large for gestational age | 1.01% (-8.78 – 11.84) | 0.85 |

The estimated effects (95% CI) are presented as a relative % change in the mtDNA content for a SD increase in maternal pre-pregnancy BMI or birth weight / compared to appropriate for gestational age newborns. Models were adjusted for newborns’ sex, gestational age, ethnicity, maternal age, household education, parity, smoking during pregnancy and cord blood thrombocyte count. n=236


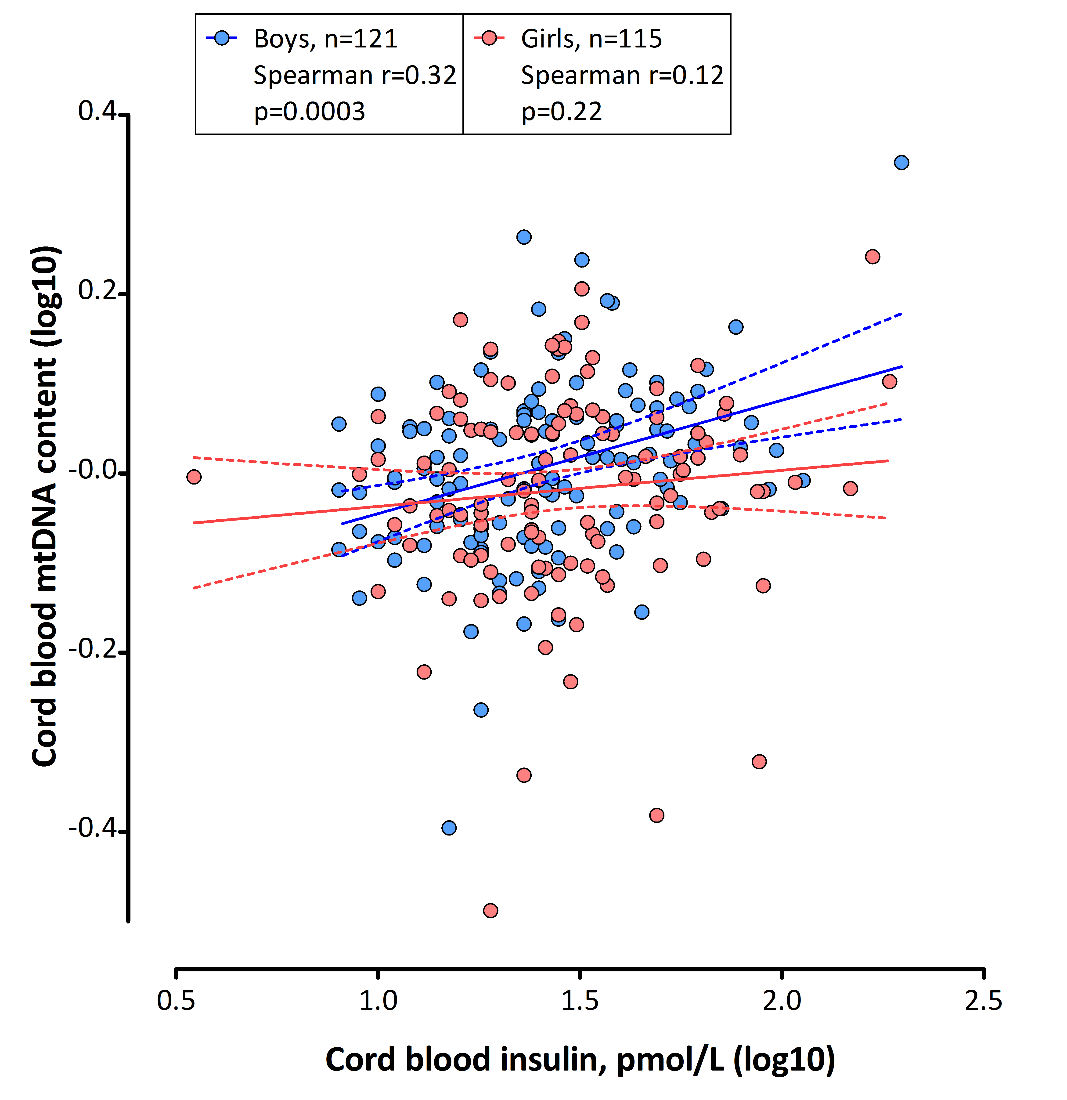


**Figure S2:** Correlation plot between cord blood insulin levels and cord blood mtDNA content, for boys (blue) and girls (orange).
